# Supplementary material for: Splice-Junction-Based Mapping of Alternative Isoforms in the Human Proteome
Source: Cell Rep. Author manuscript; Available in PMC 2020 Jan 15. (PMC6961840; doi:10.1016/j.celrep.2019.11.026)
Supplement: 2 [file NIHMS1546469-supplement-2.zip › DF1/PXD010154/Heart/Heart_14_NEXN_RAEQVNEDEENQDTAK.pdf]

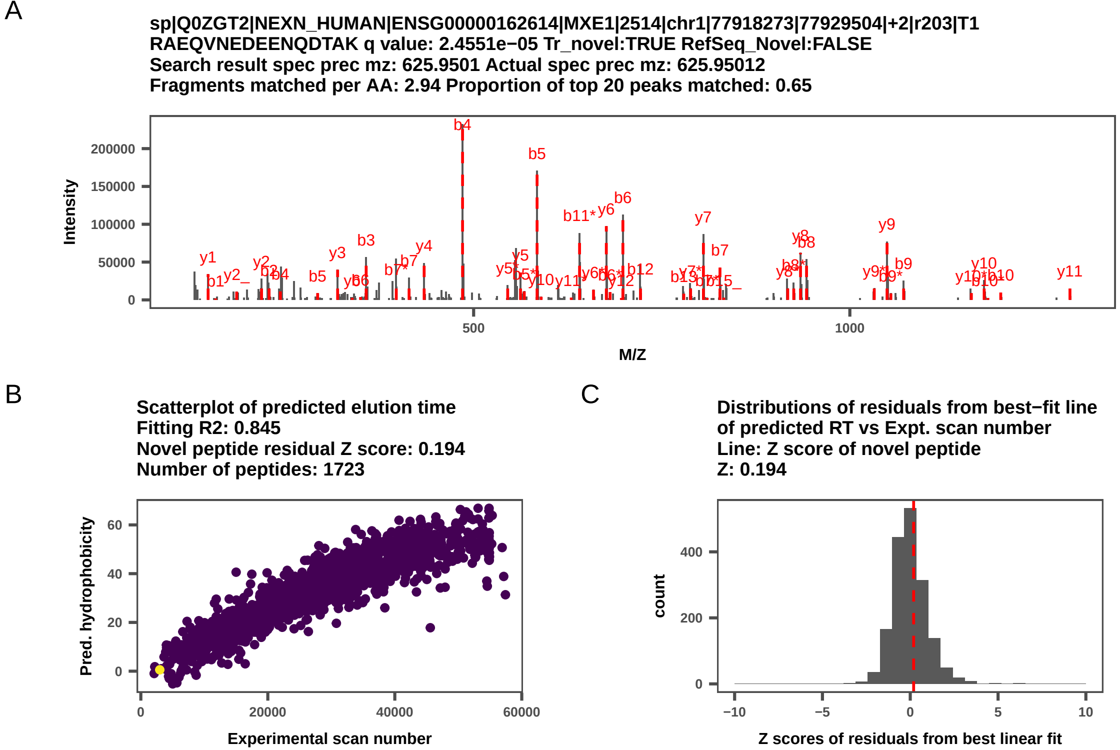
A
sp|Q0ZGT2|NEXN_HUMAN|ENSG00000162614|MXE1|2514|chr1|77918273|77929504|+2|r203|T1
RAEQVNEDEENQDTAK q value: 2.4551e−05 Tr_novel:TRUE RefSeq_Novel:FALSE
Search result spec prec mz: 625.9501 Actual spec prec mz: 625.95012
Fragments matched per AA: 2.94 Proportion of top 20 peaks matched: 0.65
Intensity
200000
150000
100000
50000
0
500
1000
M/Z
b4
b5
b6
y6
b11*
y7
y9
y8
b8
b7
y1
b1
y2
b3
y3
y4
b7
b7*
y5
y10
y11
b12
y12
b9
y10
b10
y11
B
Scatterplot of predicted elution time
Fitting R2: 0.845
Novel peptide residual Z score: 0.194
Number of peptides: 1723
Pred. hydrophobicity
60
40
20
0
0
20000
40000
60000
Experimental scan number
C
Distributions of residuals from best−fit line
of predicted RT vs Expt. scan number
Line: Z score of novel peptide
Z: 0.194
count
400
200
0
−10
−5
0
5
10
Z scores of residuals from best linear fit
